# Changes of improvement in upper limb function predict surgical outcome after laminoplasty in 1 year in patients with cervical spondylotic myelopathy: a retrospective study

**DOI:** 10.1186/s13018-023-03805-6

**Published:** 2023-04-26

**Authors:** Takuma Fudo, Ryuki Hashida, Kimiaki Yokosuka, Kimiaki Sato, Koji Hiraoka

**Affiliations:** 1 Department of Orthopaedics, Kurume University, 67 Asahi-machi, Kurume, Fukuoka 830-0011 Japan; 2 Division of Rehabilitation, Kurume University Hospital, 67 Asahi-machi, Kurume, Fukuoka 830-0011 Japan

**Keywords:** Upper limb function, Surgical outcome, Laminoplasty, Cervical spondylotic myelopathy, Simple Test for Evaluating Hand Function (STEF), Hand grip strength, Hospitalization, Japanese Orthopaedic Association (JOA) score

## Abstract

**Background:**

Cervical spondylotic myelopathy preoperative prognostic factors include age, preoperative severity, and disease duration. However, there are no reports on the relationship between changes in physical function during hospitalization and postoperative course, and in recent years, the length of hospital stay has shortened. We aimed to investigate whether changes in physical function during hospitalization can predict the postoperative outcome.

**Methods:**

We recruited 104 patients who underwent laminoplasty for cervical spondylotic myelopathy by the same surgeon. Physical functions, including Simple Test for Evaluating Hand Function (STEF), grip strength, timed up and go test, 10-m walk, and time to stand on one leg, were assessed at admission and discharge. Patients with the Japanese Orthopaedic Association (JOA) score improvement rate of 50% or more were defined as the improved group. Decision tree analysis was investigated factor for identifying improvement in the JOA score. According to this analysis, we divided into two groups using age. Then, we conducted a logistic regression analysis to identify factors that improve the JOA score.

**Results:**

The improved and non-improved groups had 31 and 73 patients, respectively. The improved group was younger (*p* = 0.003) and had better improved Δgrip strength (*p* = 0.001) and ΔSTEF (*p* < .0007). Age was significantly positively correlated with disease duration (*r* = 0.4881, *p* =  < .001). Disease duration exhibited a significant negative correlation with the JOA score improvement rate (*r* = − 0.2127, *p* = 0.031). Based on the decision tree analysis results, age was the first branching variable, with 15% of patients ≥ 67 years showing JOA score improvement. This was followed by ΔSTEF as the second branching factor. ΔSTEF was selected as the factor associated with JOA improvement in patients ≥67 years (odds ratio (OR) 1.06, 95% confidence interval (CI) 1.01–1.12, *p*=.0268); in patients <67 years, Δgrip strength was identified (OR 1.30, CI 1.04‒1.62, *p*=.0049).

**Conclusions:**

In the improved group, upper limb function improved more than lower limb function from the early postoperative period. Upper limb function changes during hospitalization were associated with outcomes one year postoperatively. Improvement factors in upper extremity function differed by age, with changes in grip strength in patients < 67 years and STEF in patients ≥ 67 years, reflecting the outcome at one year postoperatively.

## Background

Cervical spondylotic myelopathy (CSM) is a condition in which the cervical spinal canal is narrow, and age-related changes such as osteophyte formation, disk prolapse, and thickening of the yellow ligament cause pressure on the cervical spinal cord. This results in symptoms such as sensory and motor deficits in the upper extremities, lower extremities, trunk, and bladder and rectal disturbances. The initial symptoms of CSM begin with numbness in the upper extremities and impaired fine motor skills, followed by spastic paralysis of the lower extremities and thermal dysesthesia of the lower extremities [1, 2]. Surgical treatment for CSM is effective, and laminoplasty, a common procedure, is widely performed. The results of laminoplasty have been reported to be excellent [3, 4], and patient satisfaction has been high [5].

The Japanese Orthopaedic Association (JOA) score is frequently used to determine the postoperative efficacy of cervical myelopathy [6]. The JOA score comprises four subcategories: impaired hand dexterity and upper limb muscle weakness, gait disturbance, sensory disturbance, and bladder-rectal disturbance, with a minimum total score of − 2 points and a maximum score of 17. Preoperative prognostic factors for CSM have been reported to include age, preoperative severity of illness, and disease duration [7, 8]. However, there are few reports on changes in physical function during hospitalization and post-surgery.

Assessment methods for physical function in patients with cervical myelopathy include grip strength and simple upper extremity function tests (Simple Test for Evaluating Hand Function [STEF]) for upper extremity function, and gait time and timed up and go (TUG) test for lower extremity function.

STEF measures the time taken to perform a series of grasping, moving, and releasing actions on ten different objects. Therefore, STEF can capture in detail the fine motor deficits characteristic of CSM and correlate with the JOA score [9, 10]. In recent years, the length of hospital stay for spine surgery has been shortened in Japan and other countries to address medical cost issues [11, 12]. It is challenging to predict outcomes after a short hospital stay.

If we can clarify how changes in physical function during hospitalization affect post-discharge outcomes, the goals of inpatient rehabilitation can be clarified. Therefore, our study aimed to investigate the relationship between changes in physical function and outcomes during the perioperative period in patients with cervical spondylotic myelopathy.

## Methods

### Participants

**Fig. 1:**
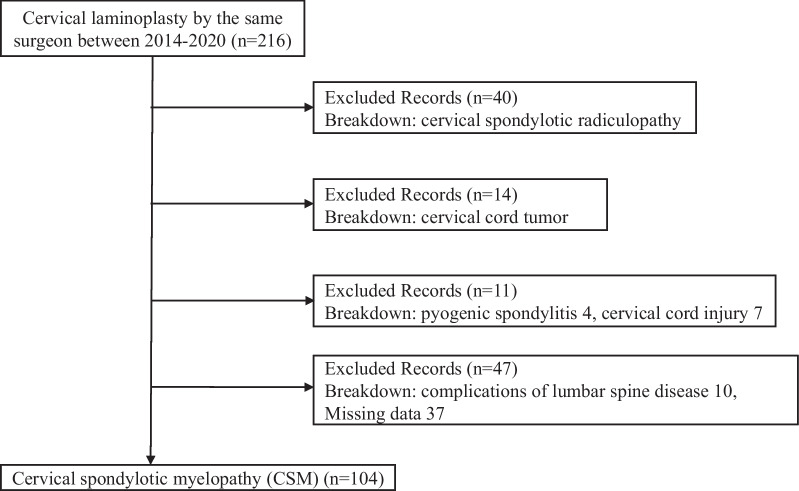
Diagram of patient's inclusion and exclusion criteria in this study

This was a retrospective study of 216 patients with cervical spine disease who had undergone laminoplasty by the same surgeon without serious complications (such as ischemic heart disease and stroke) between January 2014 and February 2021 at the Department of Orthopedic Surgery, Kurume University Hospital. The study included patients with cervical myelopathy and excluded 40 patients with cervical radiculopathy, 14 patients with tumors, four with pyogenic spondylitis, seven with cervical cord injury, and 47 with lumbar spine complications and missing data. Finally, 104 participants were included in this study (Fig. 1). In accordance with tenets of the Helsinki Declaration, the study was approved by the Kurume University Ethics Committee (Approval ID: 22025). Consent to participate in the study was obtained through an opt-out approach.

### Recorded data

The JOA score was assessed by a surgeon preoperatively and one year postoperatively. Physical function tests were assessed by a physical therapist on admission and at discharge.

### JOA

JOA score was used to determine the severity of cervical spondylotic myelopathy before and after surgery. The JOA score was scored on a 17-point scale for motor function of the upper and lower extremities, the sensory function of the upper and lower extremities and trunk, and bladder-rectal disturbances. JOA score improvement rate was defined (postoperative JOA score- preoperative JOA score)/(17 − preoperative JOA score) × 100. As in a previous report [13], JOA score improvement rate of 50% or more was defined as the improved group, while those with an improvement rate less than 50% as the non-improved group.

### STEF

**Fig. 2:**
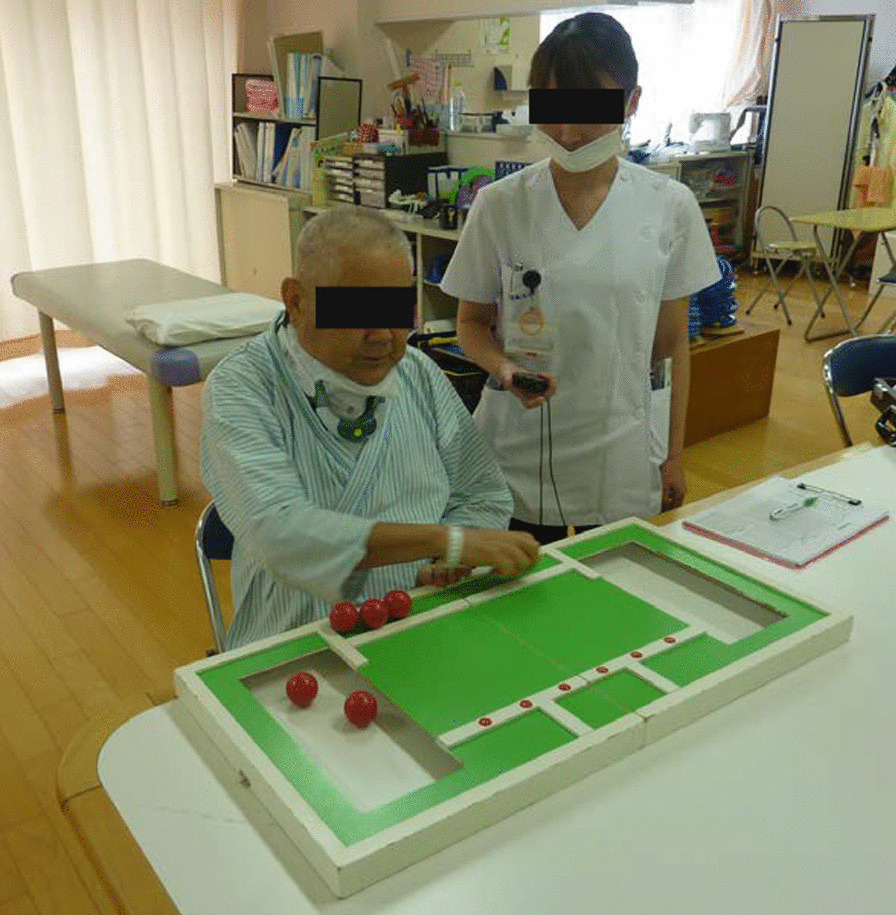
Simple Test for Evaluating Hand Function; STEF

STEF is a test developed by Kaneko et al. [14] to objectively evaluate upper limb movements by measuring the time required to move objects on a desk. The test consists of 10 subtests. The patient performs a series of grasping, moving, and releasing movements of 10 different balls or pins of different sizes, shapes, weights, and materials on a test table. Each item is scored out of 10 points according to the time required for each movement, and the total score for the ten items out of 100 points is recorded. In this study, the dominant hand was used (Fig. 2).

### Hand grip strength

Grip strength was measured using a Smedley-type grip strength meter (Takei Kiki Kogyo, T.K.K. 5101) [15]. The patient was told, “Please grip the grip strength meter as strongly as possible after the cue. Further, please hold the grip strength meter for 3 s.” The maximum value was taken twice for the dominant hand.

### TUG

TUG was performed by using a stopwatch to measure the time taken for the patient to stand from a chair to a target 3 m ahead, return to the chair, and sit. The height of the chair was 42.0 cm with an armrest. The patient was instructed to “get up from the chair, walk as fast as possible around the target 3 m ahead, and sit down in the chair again. The direction of rotation can be in either direction.” [16].

### 10-m walking test

A 10-m tape was placed on a flat floor at intervals of 10 m. In addition, tapes were placed 3 m in front of and behind the tape to provide a spare path, making a total distance of 16 m. The patient was asked to “walk toward the 16-m tape in front of you as you normally walk.” Measurement using a stopwatch started when the leading foot stepped on or over the 10-m start line and ended when the leading foot stepped on or over the 10-m end line.

### One-leg standing time

Participants were instructed to gently raise one of their lower limbs from a posture in which they held a handrail with their upper limbs with their eyes open and to stand on one leg as long as possible to measure one-leg standing time. The time until either the upper limb or pelvis touched the handrail or the raised lower limb touched the floor was measured.

### Statistical analysis

All statistical analyses were performed using JMP Version 15.0 statistical software (SAS Institute Inc., Cary, NC, USA).

Pearson correlation coefficients were used to determine the correlation between disease duration and age or JOA score improvement rate. The Pearson correlation coefficient represents the strength of the correlation and ranges from − 1 to 1. Correlation coefficient values are expressed in increments of 0.2, from very weak to very strong; 0 to 0.19 is considered very weak, either positive or negative, and 0.8–1.0 is considered very strong [17, 18].

STEF, grip strength, TUG, 10-m walk, and one-leg standing time were assessed at admission and discharge. The change (Δ) in physical items was defined as the difference from discharge to admission; the Wilcoxon rank-sum test was used to compare the two groups: the JOA score improved and the non-improved groups. In addition, a decision tree analysis was used to investigate factors associated with JOA score improvement. Age, sex, body mass index (BMI), disease duration, hospitalization period, STEF, grip strength, TUG, 10-m walk, and one-leg standing time were employed as explanatory variables.

Based on the results of the decision tree analysis, patients were divided into two groups: those ≥ 67 years and those < 67 years, and factors related to JOA score improvement were investigated in each group using logistic regression analysis. In addition, the two groups were compared for Δgrip strength and ΔSTEF in the improved and non-improved groups for the 67 years and older and those under 67 years of age. All data are expressed as median (interquartile range [IQR]) and range. In all cases, *P* < 0.05 was considered statistically significant.

## Results

### Correlation with disease duration

**Fig. 3:**
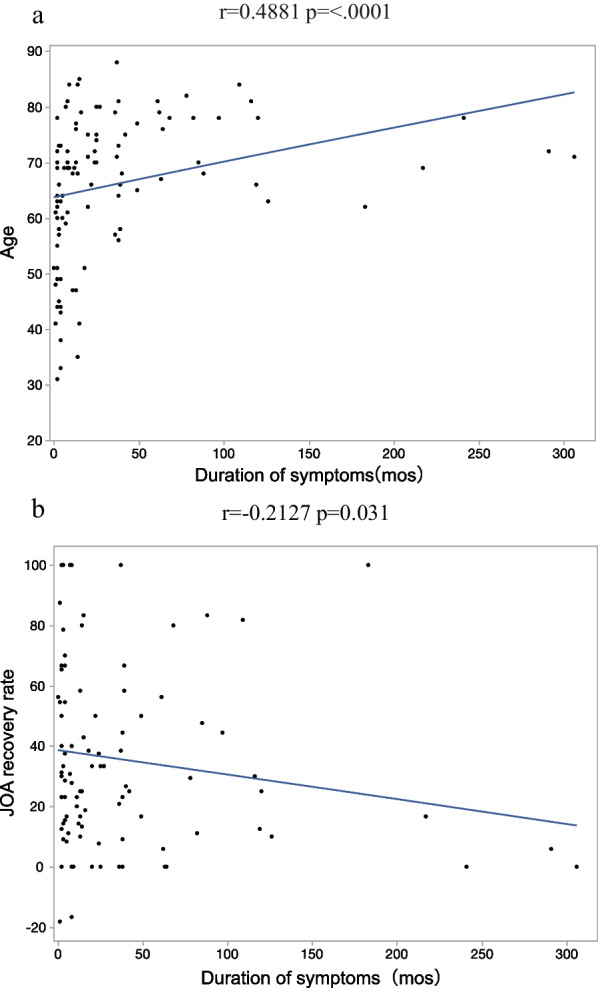
Correlation between duration of disease and age or JOA improvement rate. **a** Disease duration and age were positively correlated. **b** Disease duration and age were negatively correlated

Age had a significant positive correlation with disease duration (*r* = 0.4881, *p* < 0.001). This indicates that the older the patient, the longer the disease duration. Furthermore, there was a significant negative correlation between the JOA score improvement rate and disease duration (*r* = − 0.2127, *p* = 0.031). The longer the disease duration, the lesser the improvement in the JOA score observed (Fig. 3).

### Comparison between the improved and non-improved groups

Table 1 shows a comparison of the JOA scores of improved and non-improved groups. There were no significant differences in sex, BMI, and hospitalization period; however, the improved group was significantly younger and had a shorter disease duration. As for changes in physical parameters during hospitalization, grip strength, and STEF were significantly improved in the improved group, but there were no differences in TUG, 10-m walk, and time to stand on one leg.

**Table 1:** Comparing patient characteristics between the JOA score improved and non-improved groups

	Improved (*n* = 31)		Non-improved (*n* = 73)		*P* value
	Median	IQR	Median	IQR	
Age	62	51–69	70	63–77.5	0.003
Sex (males/females)	20/11		55/19		0.262
BMI (kg/m^2^)	23.8	19.8–25.1	23.3	20.3–26.1	0.934
Disease duration (mo)	7	2–39	19	6.3–39.5	0.0275
Hospitalization period	14	13–18	14	13–16	0.742
ΔHand grip strength (kg)	2.5	− 2.4–.5	0	− 3.8–1.9	0.001
ΔSTEF	5	1–11	1	− 1.5–4	0.0007
Δ10m walking test (s)	0	− 0.9–0	0	− 0.5–0.4	0.487
ΔTUG (s)	0	− 0.6–0	0	− 0.7–0.3	0.486
ΔOne-leg standing test (s)	0	0–9.3	0	0–1.5	0.178

*IQR* interquartile range, *BMI* body mass index, *CSM* cervical spondylotic myelopathy, *STEF* Simple Test for Evaluating Hand Function, *TUG* timed up and go test

### Factors associated with JOA score improvement in patients with cervical myelopathy over 67 years old

A multivariate logistic analysis was performed on the explanatory variables, including sex, age, disease duration, hospitalization period, BMI, Δ grip strength, Δ STEF, Δ TUG, Δ 10-m walk, and Δ time to stand on one leg.

**Fig. 4:**
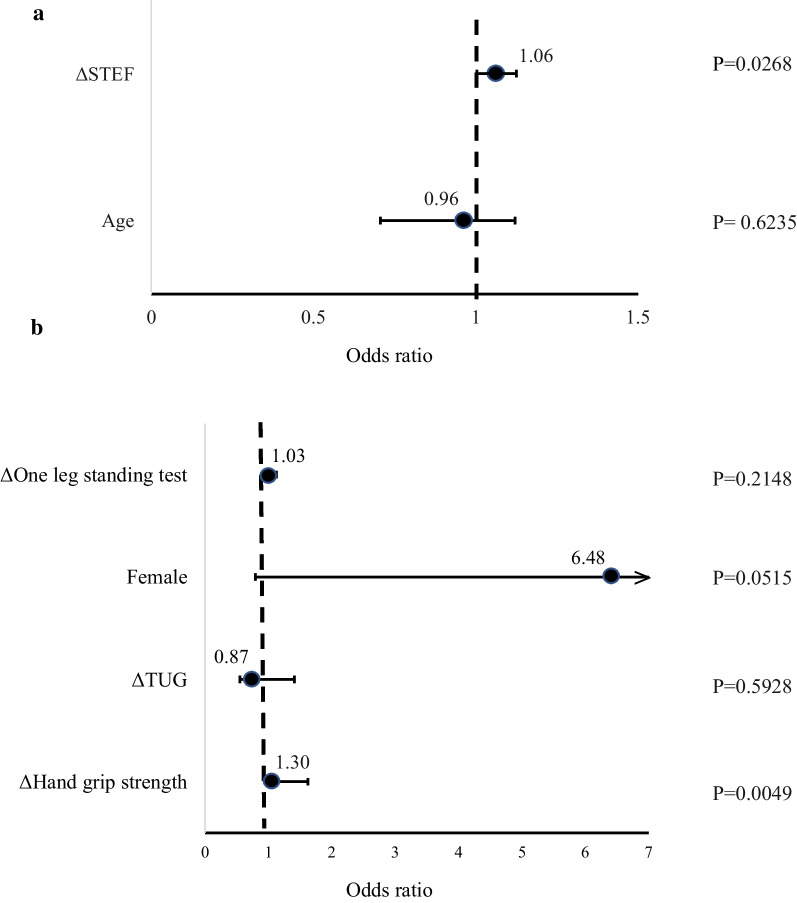
**a** The independent factor for improvement of JOA score in patients who were over 67 years. Factors associated with ΔSTEF improving JOA in 67 years and older. **b** The independent factor for improvement of JOA score in patients who were less than 67 years. Factors associated with Δgrip strength improving JOA below 67 years

Using the stepwise method, two factors, ΔSTEF and age were selected as explanatory variables. Logistic regression analysis identified ΔSTEF as a positive factor associated with improved JOA score in patients with cervical myelopathy ≥ 67 years (Fig. 4a).

### Factors associated with improvement in JOA score in patients with cervical myelopathy younger than 67 years

Using a stepwise method for the “Factors associated with improvement in JOA score in patients with cervical myelopathy under 67 years of age,” four factors were selected as explanatory variables: Δgrip strength, sex, ΔTUG, and Δone-leg stand. Logistic regression analysis identified Δgrip strength as a positive factor associated with improvement in JOA score in patients with cervical myelopathy younger than 67 years (Fig. 4b).

### Decision tree analysis algorithm to identify profiles associated with JOA score improvement

A decision tree analysis algorithm was created using three branching variables to identify profiles associated with JOA score improvement and classified into four groups.

Age was the first branching variable; 15% of patients ≥ 67 years had JOA score improvement; ΔSTEF was the second branching factor in the group ≥ 67 years; all patients with STEF improvement greater than or equal to 0 had JOA score improvement.

**Fig. 5:**
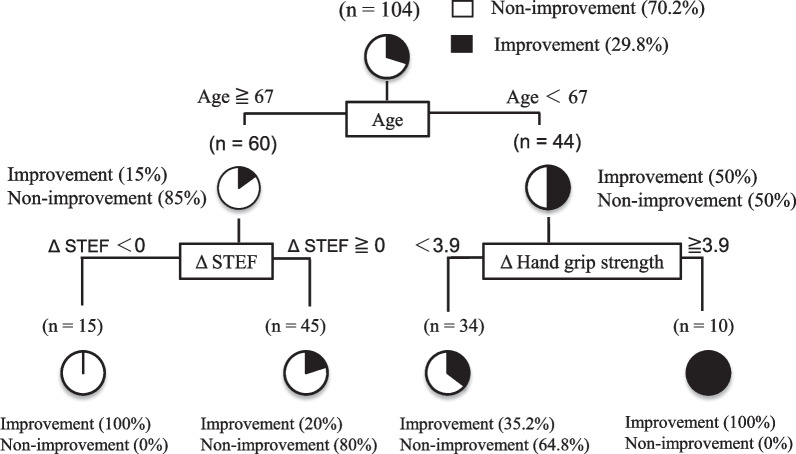
Decision tree analysis of factors involved in JOA score improvement

In contrast, 50% of the patients in the group younger than 67 years of age showed improvement in the JOA score. ΔGrip strength was the second branching factor in the group younger than 67 years of age. All patients with improved grip strength by more than 3.9 kg showed improvement in the JOA score (Fig. 5).

### Comparison of improved and non-improved groups for Δgrip strength and ΔSTEF at age 67 years and older and at age < 67 years

Table 2 shows the comparison between the improved and non-improved groups for Δgrip strength and ΔSTEF at age 67 years and older and the comparison between the improved and non-improved groups at age less than 67 years. The results for both items were significantly different for those under 67 years of age (*p* = 0.0019) and for ΔSTEF (*p* = 0.0085).

**Table 2:** Comparison of STEF and grip strength of patients ≥ 67 to those of patients < 67 years of age

	Improved (*n* = 9)		Non-improved (*n* = 51)		*P* value
	Median	IQR	Median	IQR	
*≥ 67 years*					
ΔHand grip strength (kg)	2.3	0.25–6.7	0.2	− 1.2–2.0	0.0855
ΔSTEF	12	3.5–16	1	− 2.0–4.5	0.0041

	Improved (*n* = 22)		Non-improved (*n* = 22)		*P* value
	Median	IQR	Median	IQR	
*< 67 years*					
ΔHand grip strength (kg)	2.8	0–5.9	0	− 3.2–1.7	0.0019
ΔSTEF	5	1.0–10.5	0	− 1.0–4.0	0.0085

*IQR* interquartile range, *STEF* Simple Test for Evaluating Hand Function

## Discussion

This study investigated the association between perioperative changes in physical function and outcomes in cervical spondylotic myelopathy. Changes in upper extremity function during hospitalization were associated with outcomes at one year postoperatively. Based on the decision tree analysis, patients were divided into two groups, those aged 67 years or older and those younger than 67 years. Multivariate logistic analysis showed that ΔSTEF was associated with improvement in JOA score in patients aged 67 years or older, and Δgrip strength was associated with improvement in JOA score in those younger than 67 years. Thus, we found that the factors associated with improvement in upper limb function differed by age. The perioperative upper limb function of CSM was found to reflect the amount of change in grip strength in patients younger than 67 years and the amount of change in STEF in patients ≥ 67 years at one year postoperatively.

### Consideration 1: correlation with the duration of disease

In this study, a significant correlation was observed between the duration of disease and age and the rate of JOA score improvement. In other words, the older the patient is, the longer the disease duration tends to be. Nagata et al. reported that the disease duration before surgery was significantly longer in elderly patients than in younger patients [19]. There have been many other reports on the effect of disease duration on the postoperative outcome of JOA score improvement in elderly patients who underwent laminoplasty for CSM [18, 20]. There are also reports that the poor improvement of postoperative symptoms in elderly patients is due to age-related changes in the spinal cord itself, such as a decrease in anterior horn cells and myelinated fibers [21, 22]. This study is similar to previous reports, suggesting that spinal cord degeneration in elderly patients may progress and become irreversible as the disease duration increases and that this may lead to a condition in which postoperative symptom improvement is not expected.

### Consideration 2: immediately after surgery, upper limb function improves faster than lower limb function.

The results of the study showed that perioperative physical function of the upper limb (grip strength and STEF) was significantly improved in the JOA score improved group compared to the non-improved group (Table 2). The average length of stay in our hospital was 15.0 days. In other words, upper extremity function tended to improve earlier than lower extremity function at approximately two weeks postoperatively in the improved group. In a previous study, Fujiwara et al. reported that STEF, which indicates hand motor function, improved from 1 week postoperatively [9]. Hosono et al. [23] performed laminoplasty for cervical spondylotic myelopathy and reported that the 15-s grip-and-release test, a measure of fine motor function, increased rapidly within 24 h after surgery. Prabhu et al. also focused on the Rapid Hand Flick Test, which evaluates fine motor skills, and found improvement from day one postoperatively in CSM [24]. Sakai et al. found that grip strength improved from 3 months postoperatively in both right and left hands [25].

Thus, although the period of improvement and upper extremity endpoints varied among the articles, upper extremity physical function (STEF and grip strength) seemed to improve from the early postoperative period.

Regarding lower extremity function, a significant improvement in TUG was reported from 8 weeks after laminoplasty [26]. There is also a report of significantly faster gait speed at 12 weeks postoperatively [27]. The reason for the delayed recovery of postoperative lower extremity function compared to upper extremity function is that the pyramidal tracts innervating the lower extremity in the cervical spinal cord are poorer than those innervating the upper extremity, and lower extremity function tends to drop. Hence, postoperative recovery of lower limb function is said to take a longer period [28]. In this study as well, upper limb function tended to improve earlier than lower limb function as a change from hospitalization to discharge, and the group with improved upper limb function tended to have significantly improved JOA at one year postoperatively.

### Consideration 3: improvement factors differ depending on age

In this study, we investigated the relationship between changes in physical function and treatment outcomes during the perioperative period of CSM. During hospitalization, changes in upper limb function during the first two weeks postoperatively were associated with results at 1 year postoperatively, and factors related to improvement differed between younger and older patients. Based on the decision tree analysis of factors associated with improvement in postoperative JOA scores, ΔSTEF was a divergent factor for patients 67 years and older and Δgrip strength for those younger than 67 years. Based on these results, a multivariate logistic analysis of the JOA score in patients 67 years of age and older and those younger than 67 years of age revealed that ΔSTEF and Δgrip strength were factors associated with JOA score improvement in patients 67 years of age and older and those younger than 67 years of age, respectively. These results suggest that the amount of change in STEF during hospitalization was identified as a factor for improving JOA score at one year postoperatively in patients with CSM in those ≥ 67 years, while the amount of change in grip strength in those younger than 67 years was identified as a factor for improving JOA score at one year postoperatively.

Thus, this study revealed that postoperative improvement factors differed by age.

The duration of observation may have affected the results since this study investigated improvement factors at approximately two weeks postoperatively.

Generally, muscle hypertrophy is obtained 4–6 weeks after the start of training [29, 30]. However, grip strength has been reported to improve after 12 weeks of a training intervention in elderly patients [31, 32], and muscle hypertrophy is said to take longer than in younger patients [33].

Suzuki et al. [34] reported that grip strength improved in elderly patients with cervical myelopathy six months after surgery, and it takes a longer time for grip strength to improve in both normal patients and patients with cervical myelopathy.

Behind the selection of ΔSTEF as an improvement factor in patients aged 67 years or older, the short observation period of approximately two weeks after surgery may have affected the results since muscle hypertrophy takes a longer time in elderly patients.

In other words, although the cause of this remains unclear, the study investigated the relationship between changes in physical function during the perioperative period and postoperative outcomes, and it became clear that the items of upper extremity function that should be focused on differ according to age.

### Limitations

This study has some limitations. First, this was a single-center, backward-looking study. Future prospective studies on physical function should be conducted. Second, the JOA score is a subjective method of evaluation on the part of the healthcare providers, and it is necessary to evaluate it using more subjective and objective indices in the future. Third, we didn’t take into account the relationship categories of STEF and surgical outcome. Fujiwara et al. reported that the preoperative evaluation of “coordinated motion” of the STEF was significantly low in the non-improved group [9]. We didn't analyze the categories of STEF in this study. Thus, further prospective study with categories of STEF is required to elucidate the relationship between surgical outcomes.

## Conclusion

We investigated the relationship between changes in physical function and outcomes in the perioperative period of cervical spondylotic myelopathy. In the improved group, upper limb function tended to improve more than lower limb function in the early postoperative period, and changes in upper limb function during hospitalization were associated with outcomes at one year postoperatively. In addition, the factors of improvement in upper extremity function differed by age, with the change in grip strength in patients younger than 67 years and the change in STEF in patients ≥ 67 years reflecting performance at one year postoperatively.

## Data Availability

The datasets generated during and/or analyzed during the current study are available in the Harvard Dataverse repository, 10.7910/DVN/AFFUC5 [35].

## Abbreviations

JOA: Japanese Orthopaedic Association
STEF: Simple Test for Evaluating Hand Function
TUG: Timed up and go test
CSM: Cervical spondylotic myelopathy
BMI: Body Mass Index
