# Correction: Changes of improvement in upper limb function predict surgical outcome after laminoplasty in 1 year in patients with cervical spondylotic myelopathy: a retrospective study

**DOI:** 10.1186/s13018-024-04568-4

**Published:** 2024-02-01

**Authors:** Takuma Fudo, Ryuki Hashida, Kimiaki Yokosuka, Kimiaki Sato, Koji Hiraoka

**Affiliations:** 1 Department of Orthopaedics, Kurume University, 67 Asahi-machi, Kurume, Fukuoka 830-0011 Japan; 2 Division of Rehabilitation, Kurume University Hospital, 67 Asahi-machi, Kurume, Fukuoka 830-0011 Japan

**Correction: Journal of Orthopaedic Surgery and Research (2023) 18:323** 10.1186/s13018-023-03805-6

Following publication of the original article [1], the abstract section was incorrectly given as “ΔSTEF was selected as the factor associated with JOA improvement in patients ≥ 67 years (odds ratio (OR) 0.95, 95% confidence interval (CI) 0.90–0.99, p = 0.047); in patients < 67 years, Δgrip strength was identified (OR 0.53, CI 0.33‒0.85, p = 0.0086).”, but should have been “ΔSTEF was selected as the factor associated with JOA improvement in patients ≥ 67 years (odds ratio (OR) 1.06, 95% confidence interval (CI) 1.01–1.12, p = 0.0268); in patients < 67 years, Δgrip strength was identified (OR 1.30, CI 1.04‒1.62, p = 0.0049).”

The authors identified an error in Fig. 4. The correct Fig. 4 is given below.

**Fig. 4:**
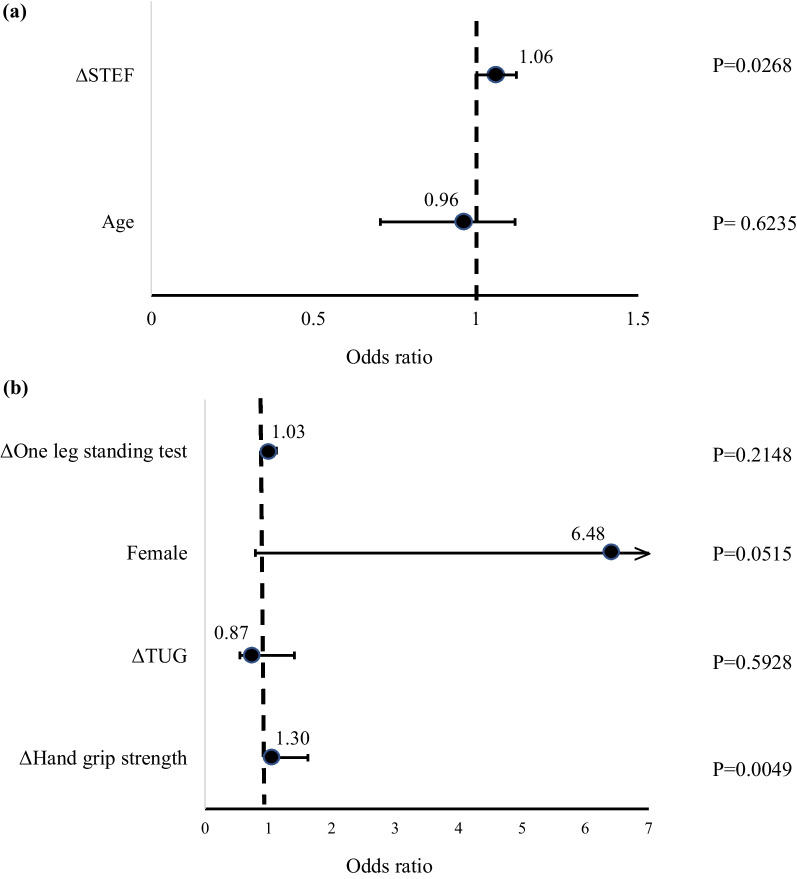
**a** The independent factor for improvement of JOA score in patients who were over 67 years. Factors associated with ΔSTEF improving JOA in 67 years and older. **b** The independent factor for improvement of JOA score in patients who were less than 67 years. Factors associated with Δgrip strength improving JOA below 67 years

The original article [1] has been corrected.
